# Supplementary material for: Fluorescent detection of PARP activity in unfixed tissue
Source: PLoS One. 2021 Jan 22;16(1):e0245369. doi: 10.1371/journal.pone.0245369 (PMC7822349; doi:10.1371/journal.pone.0245369)
Supplement: Attachment — Submitted filename: Response to Reviewers.docx [file pone.0245369.s001.docx]

**Reviewer 1:**

**The present study by Belhadj et al. describes a new assay for detection of PARP activity, which is easier to perform than previously reported two-step procedures. It requires only one step and is demonstrated to work on non-fixed tissue.**

**In order to be useful for the scientific community, the used reagents need to be made available, either by describing the procedures for their preparation and their analytical data or (I understand that two of the authors are employees of a company called Biology, which is specialized in selling nucleotide derivatives), if the company may hesitate to describe the synthesis, needs to be made commercially available. For the biotinylated derivative this seems to be the case (?, see p. 5, PARP in situ activity assays, also for the fluorescent probe, p.5, l. 111), but it would be good to state this clearly in the manuscript.**

Response: We thank the reviewer for this suggestion. We note that the all the reagents used in the study are indeed commercially available. However, we may not have expressed this clearly enough. To improve clarity, we have rephrased it in the Materials & Methods and added their catalogue numbers (l. 107, 114, and 115)

**The fluorescent, as well as the biotinylated analog seem to work nicely in unfixed tissue samples, albeit all of them look rather cell impermeable. I could not detect any permeabilization step in preparation of the tissue samples, is this a specific property of the retinal samples? Also the antibody incubation works nicely, so permeability of the tissue is obviously enhanced. The authors should comment here.**

Response: There is indeed no separate permeabilization step. However, the PARP reaction mixture contains Triton X100 (l. 107 and 115). Regarding the immunostaining, PBS with 0,3% Triton X100 was used in several steps of the protocol: quenching, blocking and incubations with both primary and secondary antibodies (l. 122, 123 and 125).

**Is it possible to determine the KD of the used probes to PARP? The conclusion says that the sensitivity of the one-step assay is somewhat lower (p.15, l 338), the introduction claims the sensitivity of the two assay systems to be essentially the same (p.10, l. 79). This should be homogenized. The point of decreased sensitivity is possibly not completely correct, as fluorescein is not an extremely bright and photostable dye (and as the authors state correctly, the antibody two-step protocol also offers the advantage of signal amplification), so sensitivity of detection is lower, but sensitivity in terms of interacting with PARP may still be very high (just the antibody format employing multiple dyes).**

Response: We have not determined the K_D_ values for the different NAD^+^ analogues. In the original submission there was indeed a discrepancy between what we said about probe sensitivity in introduction and conclusion. The reason for this was exactly as described by the reviewer, the lack of an amplification step in the single-step assay may reduce the apparent sensitivity, even though the binding affinities of the two PARP probes may in fact be very similar. We have now rephrased this aspect and harmonized the statements made in introduction (l. 80-83), discussion (l. 296-303), and conclusion (l. 352-354).

**Is there an explanation for the different location of fluorescent cells in Figure 2 and 4? Panel a (biotin) and panel b look quite different. This should be explained.**

Response: In Figure 2 (A, B) and Figure 4 (A, C) the results of the different PARP activity assays are shown. We did not observe a systematically different localization of PARP activity staining between 6-Biotin-17-NAD+ and 6-Fluo-10-NAD^+^. Nevertheless, when compared to the PAR immunostaining, which essentially shows only a nuclear staining in the outer nuclear layer (ONL; Figure 3), the NAD^+^ based activity assays both show an additional staining of the photoreceptor synapses in the outer plexiform layer (OPL). Since the fluorescent labelling was completely abolished by the PARP inhibitor olaparib (Figure 4), both the nuclear as well as the synaptic label are very likely PARP specific.

To account for the reviewer’s comment, we have changed the images shown in figure 4 to more accurately represent the staining obtained with the different NAD^+^ analogues.

**The conclusion could still discuss options for further improvement of the current probes in terms of reaching permeability in live cell samples – which would represent another big advantage over the current two-step protocol.**

Response: We thank the reviewer for this suggestion. Unfortunately, it may be difficult to change the structure of the molecule without affecting its PARP substrate properties. We now present this difficulty in the discussion (l. 342-347) and suggest as alternative solution the encapsulation in a drug delivery system such as liposomes or nanoparticles.

**Minor point: p.15, l. 311 says Ref. Paquet-Durand 2007, please update.**

Response: We thank the reviewer for pointing this out. The reference has been updated (l. 320).

**In conclusion, the present manuscript needs some minor clarification and addition but the contained data is valuable, well presented and should be published once the points raised above have been addressed.**

**Reviewer 2:**

**The manuscript by Belhadj et al describes the implementation of a commercially available fluorophore substrate for PARP, provided by the company where the authors are employed.**

Response: Indeed, two of the co-authors are working at Biolog, a company that, among other compounds, produces and sells NAD^+^ analogues. This was clearly stated in the conflicts of interest section and is also obvious from the author affiliations. The fact that the used NAD^+^ analogues are from the company Biolog was also specified in the materials and methods section. Nevertheless, to address the reviewer´s concern, this has now been highlighted even further by giving the respective catalog numbers.

**The work deals with testing 6-Fluo-10-NAD in ex vivo retinal sections, comparing to established epsilon NAD and a biotinylated NAD. While the authors show that the signal produced by 6-Fluo-10-NAD in ex vivo sections is similar to that using the biotinylated compound, the novelty of this work is limited. The authors fail to report on other published methods for ex vivo PARP activity labelling in tissues (doi.org/10.1021/acs.bioconjchem.9b00089).**

Response: We thank the reviewer for this suggestion. The suggested publication is now included in the discussion (l. 291-294).

**They also fail to fully demonstrate the proportion of PAR positive cells stained by 6-Fluo-10-NAD. While immunocytochemistry was performed to illustrate PAR staining, it was done on different sections than the ones stained by 6-Fluo-10-NAD. As previously demonstrated (doi.org/10.1021/acs.bioconjchem.9b00089), the same sections should have been stained with both 6-Fluo-10-NAD and anti-PAR antibody for analysis by immunofluorescence microscopy. Otherwise, specificity is not exhaustively shown as claimed. Considering that fixation was performed after staining, it is not clear why this couldn't have been done.**

Response: We thank the reviewer for this question. The fixation of tissue samples would destroy the activity of the PARP enzyme, hence, it is essential to use unfixed tissue for the PARP activity assay. On the other hand, the immunostaining for PAR necessitates PFA fixed tissue. This is why a co-localization of PARP activity assay and PAR immunostaining is not easily done.

Nevertheless, in response to this comment, we attempted to perform the co-localization as requested, on the same sections, stained both with 6-Fluo-10-NAD^+^ (and, as additional control, also with 6-Biotin-17-NAD^+^) and anti-PAR antibody. To this end, we used unfixed tissue to first perform the PARP activity assay, then we fixed the tissue section with 4% PFA, and subsequently performed PAR immunostaining on that same section. As controls, we used the individual staining procedures performed separately on unfixed and fixed tissue, respectively. The results of these stainings are shown in the Figure 1 below, which we provide for review purposes only.

In control situations, the PARP activity assay revealed numerous positive cells (Figure 1A). Likewise, the immunostaining for PAR revealed positive cells in the outer nuclear layer (ONL; Figure 1B). However, when PARP activity assay was performed first and then followed by fixation, quenching of endogenous peroxidase activity, PAR staining, and numerous washing steps, PARP activity label was no longer detectable (Figure 1C). Conversely, the PAR staining, when performed on unfixed sections, which were then fixed only after the PARP activity assay had been performed, displayed a strong background and no positive label (Figure 1D).

**Figure 1: Colocalization of PARP Activity and PAR DAB Immunostaining.** In the control panel (left), the PARP activity assay was performed on unfixed tissue sections as described in the manuscript (**A**), while the PAR DAB staining was performed on fixed tissue sections (**B**). In the co-localization panel (right) (**C**, **D**), the PARP activity assay was performed first on unfixed tissue sections, then, these were fixed with 4% PFA, before performing the PAR DAB staining. Note that PARP activity was undetectable in the co-localization experiment, while PAR staining delivered a very high background without specific labeling. DAPI (grey) was used as nuclear counterstaining in A and C. ONL = outer nuclear layer, INL = inner nuclear layer, GCL = ganglion cell layer.

**Additionally, the value of the method presented is unclear: After staining with 6-Fluo-10-NAD, the authors still fix the tissue prior to imaging. Why do we need to stain before fixation then? While there are claims that this approach preserves the fraction of PAR from PARG-mediated degradation, there is no demonstration that pre- versus post-fixation labelling has any impact on PAR compliment.**

Response: Here, there seems to be a misunderstanding. The PARP activity assay is, in fact, performed on unfixed tissue sections, which are then directly mounted for microscopic imaging, without the need for fixation.

Moreover, we did not make claims as to the effects of pre- or post-fixation on PAR immunodetection. However, judging from the co-localization study performed for this revision (see Figure 1 above), we may now say that post-fixation is not suitable for PAR immunodetection.

**The paper states that a two-way ANOVA was used for PARP activity assay evaluation, but the need for two-way versus one-way with posthoc evaluation is unclear, and the validity of the analysis is uncertain.**

Response: We believe the 2-way ANOVA analysis is important because we are interested not only in the effects of the NAD^+^ analogues but also in the effects on the different groups (wt *vs*. *rd1*) and, more importantly, on the interactions between NAD^+^ analogues and groups. Nevertheless, we simplified the analysis by excluding the ε-NAD+ group (which was essentially 0) and confirmed the model’s perquisites for normality of the residuals. We have revised the corresponding part of the materials and methods section to better explain the statistical analysis (l. 168-170).

**The authors also state that this work may open avenues for detecting PARP activity in vivo. Again, this has already been done (doi.org/10.1021/acs.bioconjchem.9b00089).**

Response: We thank the reviewer for pointing us to this very interesting paper. In Shuhendler *et al*., the authors developed a novel radioactive probe for in vivo imaging of PARP activity using positron emission tomography. The probe was tested in cancer xenograft mouse models.

As opposed to the above study, we are interested in developing a fluorescent probe that can be combined with light-based, single-cell imaging techniques. As such the probe should be detectable both with conventional fluorescence microscopy and possibly also with current non-invasive *in vivo* imaging techniques such as adaptive optics scanning laser ophthalmoscopy. We now briefly present this concept in the discussion of the manuscript (l.334-338)

A further important difference to the Shuhendler et al., technique is that our assay does not require radioactive label nor PET detection, making it simpler and cheaper to handle for most laboratories.

**Overall, this manuscript appears to be more of a white paper, describing the implementation of a commercial dye for an assay that is already established. The case for adopting a one-step versus two-step method is not justified, and the claimed specificity is not directly or robustly demonstrated. The work does not add new knowledge or capability to the PARP biochemistry field.**

Response: We thank the reviewer for raising this point, but we obviously beg to differ. To the very best of our knowledge, the assay using 6-Fluo-10-NAD^+^ has not previously been established for single cell PARP activity detection. Moreover, the single-step assay is faster and easier to perform, and can be implemented with standard lab equipment, allowing for a more rapid adoption by other laboratories. In the revised manuscript, we have now tried to more clearly highlight the advantages of the new *in situ* PARP activity assay (l. 189-191; l.267-270; l.351-358).
