# Supplementary material for: Fluorescent detection of PARP activity in unfixed tissue
Source: PLoS One. 2021 Jan 22;16(1):e0245369. doi: 10.1371/journal.pone.0245369 (PMC7822349; doi:10.1371/journal.pone.0245369)
Supplement: Attachment — Submitted filename: Response to Reviewers_2 (2).docx [file pone.0245369.s002.docx]

**Reviewer #2: I appreciate the care the authors have taken to address the points raised in the previous review. While most responses are justified, one outstanding issue remains: the colocalization of 6Fluo10NAD+ with PAR. While the authors state there is no fixation performed after 6Fluo10NAD staining, it is indicated in the methods in section "Testing Fluorescent NAD On Live Retina" that staining is done on P11, then on P12 tissues are fixed in 4% PFA and processed for imaging. If this is the case, then indeed immunofluorescence localization of PAR as indicated in the literature (e.g. in the work doi.org/10.1021/acs.bioconjchem.9b00089 cited) can be performed simultaneously. It is still not clear why this cannot be performed. While it is appreciated that the authors performed extra experiments trying to image PAR by DAB, the results are unconvincing. PAR staining is performed in the literature with regularity using commercial reagents. The value of 6Fluo10NAD for imaging PARP1 activity must be correlated with PAR staining, otherwise the signal of NAD utilization can be attributed to any number of other enzymes, both in the PARP family and in other families.**

Response: We appreciate the reviewer’s thoroughness very much, however, here we think there is a misunderstanding relating to the different types of experiments performed in our study. To assess PARP activity in degenerating retina, we used three different experimental conditions: 1) unfixed (frozen) retinal tissue sections; 2) PFA-fixed retinal tissue sections; and 3) live retinal explant cultures, which were also fixed with PFA.

6-Fluo-10-NAD^+^ was first used on unfixed, frozen tissue sections (see the “Histology” section of the Material & Methods for a description of the preparation of the tissue). Since the PARP enzyme cannot “survive” PFA fixation, the PARP activity assay was always conducted on unfixed sections (see the “PARP *in situ* activity assays” section of the Material & Methods). PARP activity specific signal could readily be revealed by 6-Fluo-10-NAD^+^ on such unfixed tissue sections (see the Results section “Using NAD^+^ analogues to probe for *in situ* PARP activity” and Figure 2).

In addition, 6-Fluo-10-NAD^+^ was used on ***live*** organotypic retinal explants cultures (see the Materials & Methods section “Testing fluorescent NAD^+^ on live retina”). 6-Fluo-10-NAD^+^ was added to the culture medium at a concentration of 100µM and for an incubation time of 24h after which the cultures were stopped by PFA-fixation. However, no PARP activity signal could be seen (see Results section “Testing NAD^+^”). We think that 6-Fluo-10-NAD^+^ was either not able to penetrate into the tissue or could not reach across the intact cell membranes of live cells (see Discussion section “Tissue penetration and permeability of 6-Fluo-10-NAD^+^”).

Taken together, it was not possible to colocalize PARP activity and PAR immunostaining on sections from the organotypic retinal explant cultures, because in that situation our probe did not detect PARP activity. In the previous revision, we provided the result of our trial for colocalizing PARP activity and PAR immunostaining on unfixed dead tissue sections and provided an explanation on why we think it did not work.

To address the reviewer’s question and to further improve the manuscript, we have now inserted an additional comment in the materials and methods, at the beginning of the histology section (page 5, lines 94-96).
